# Effect of Intravenous Lidocaine on Inflammatory and Apoptotic Response of Ischemia-Reperfusion Injury in Pigs Undergoing Lung Resection Surgery

**DOI:** 10.1155/2021/6630232

**Published:** 2021-06-04

**Authors:** Andrea Romera, María Cebollero, Bárbara Romero-Gómez, Francisco Carricondo, Sara Zapatero, Uxío García-Aldao, Lorena Martín-Albo, Javier Ortega, Elena Vara, Ignacio Garutti, Carlos Simón

**Affiliations:** ^1^Department of Anesthesiology, Gregorio Marañón University Hospital, Madrid, Spain; ^2^Department of Anatomic Pathology, Gregorio Marañón University Hospital, Madrid, Spain; ^3^Department of Immunology, Ophthalmology and Otorhinolaryngology, Faculty of Medicine, Complutense University of Madrid, Madrid, Spain; ^4^Department of Thoracic Surgery, Gregorio Marañón University Hospital, Madrid, Spain; ^5^Department of Biochemistry and Molecular Biology III, Faculty of Medicine, Complutense University of Madrid, Madrid, Spain

## Abstract

**Background:**

Ischemia-reperfusion injury is one of the most critical phenomena in lung transplantation and causes primary graft failure. Its pathophysiology remains incompletely understood, although the inflammatory response and apoptosis play key roles. Lidocaine has anti-inflammatory properties. The aim of this research is to evaluate the effect of intravenous lidocaine on the inflammatory and apoptotic responses in lung ischemia-reperfusion injury.

**Methods:**

We studied the histological and immunohistochemical changes in an experimental model of lung transplantation in pigs. Twelve pigs underwent left pneumonectomy, cranial lobectomy, caudal lobe reimplantation, and 60 minutes of graft reperfusion. Six of the pigs made up the control group, while six other pigs received 1.5 mg/kg of intravenous lidocaine after induction and a 1.5 mg/kg/h intravenous lidocaine infusion during surgery. In addition, six more pigs underwent simulated surgery. Lung biopsies were collected from the left caudal lobe 60 minutes after reperfusion. We conducted a double study on these biopsies and assessed the degree of inflammation, predominant cell type (monocyte-macrophage, lymphocytes, or polymorphous), the degree of congestion, and tissue edema by hematoxylin and eosin stain. We also conducted an immunohistochemical analysis with antibodies against CD68 antigens, monocyte chemoattractant protein-1 (MCP-1), Bcl-2, and caspase-9.

**Results:**

The lungs subjected to ischemia-reperfusion injury exhibited a higher degree of inflammatory infiltration. The predominant cell type was monocyte-macrophage cells. Both findings were mitigated by intravenous lidocaine administration. Immunohistochemical detection of anti-CD68 and anti-MCP-1 showed higher infiltration in the lungs subjected to ischemia-reperfusion injury, while intravenous lidocaine decreased the expression. Ischemia-reperfusion induced apoptotic changes and decreased Bcl-2 expression. The group treated with lidocaine showed an increased number of Bcl-2-positive cells. No differences were observed in caspase-9 expression.

**Conclusions:**

In our animal model, intravenous lidocaine was associated with an attenuation of the histological markers of lung damage in the early stages of reperfusion.

## 1. Introduction

Ischemia-reperfusion injury (IRI) is one of the most critical phenomena in organ transplantation [1] and directly affects survival [2]. In lung transplantation, IRI causes primary graft failure, which is one of the most frequent causes of early mortality, and it may also induce rejection, which is the principal cause of mortality after transplantation [3, 4]. During the ischemia period, cellular—including alveolar macrophages, lymphocytes, and neutrophils—and molecular mediators are activated and initiate processes of inflammation and apoptosis, potentiated upon organ reperfusion. Several biomarkers have allowed for characterization of this pathophysiological response. There are numerous therapeutic strategies to attenuate the lung damage produced by IRI. Local anesthetics (LA) modulate inflammatory cascades and have a protective effect against IRI of the heart and liver [5–7]. Although scarce, some studies have evaluated the efficacy of lidocaine in lung damage caused by IRI [8, 9] but, to date, there is no evidence of how this drug acts histologically in this clinical context.

We hypothesize that the activation of cellular and molecular mediators in lung IRI could manifest in very early stages of reperfusion through histological and immunohistochemical changes. Furthermore, possible beneficial effects of intravenous lidocaine could be evidenced in the modification of these changes. The aim of this study is to evaluate the histological and histopathological immunohistochemical changes in the early stages of reperfusion and the possible differences that can occur when administering intravenous lidocaine during a surgical procedure.

## 2. Methods

The study was carried out at the Animal Facility and Experimental Surgery Unit of Gregorio Marañón University Hospital in Madrid (Spain). Ethical approval for this study (Ethical Committee N° 64/10) was provided by the Animal Research and Experimentation Committee of the institution (Chairperson Doctor Fernando Asensio) on 12 January 2010. A total of 18 Large-White pigs, either sex, were equally divided into three groups. In the control group (CON), an orthotopic pulmonary autotransplantation was performed by means of left pneumonectomy, lobectomy ex situ, reimplantation of the caudal lobe, and reperfusion for 60 minutes. The same protocol was performed on the lidocaine group (LIDO), but an intravenous bolus of lidocaine (Braun Medical SA, Barcelona, Spain) was also administered at 1.5 mg/kg after induction, followed by continuous infusion at 1.5 mg/kg/h.

The third group was the simulated surgery or the SHAM group, in which thoracotomy was performed with bipulmonary ventilation at all times. During the procedure, different hemodynamic parameters and arterial blood studies were analyzed. After 60 minutes of reperfusion of the transplanted lobe, pulmonary biopsies were performed for histological and immunohistochemical studies.

### 2.1. Surgical Procedure

The animals were subjected to 18 hours of fasting for solids but were able to drink water until up to 20 minutes before the intervention. At 20 minutes before the induction, they were premedicated with 20 mg/kg of intramuscular ketamine (Ketalar, Parker Davis, Hameln, Germany) and 0.04 mg/kg of intramuscular atropine (Braun Medical, Barcelona, Spain). Subsequently, a vein in the ear was canalized (Introcan Safety, Braun, Germany, number 22), and the animal was monitored with continuous electrocardiogram, pulse oximetry, and an Ohmeda capnography 5250 RGM (General Electric Health Care, United States). A Servo Ventilator 900 C (Siemens), a blood gas analyzer GEM Premiere 5000, and a Pulse Index Continuous Cardiac Output (PICCO) device (Edwards, Irving, California, United States) were used. Anesthesia was induced with 2.5 *μ*g/kg of intravenous fentanyl (Fentanest, Kern Pharma, Barcelona, Spain), 2 mg/kg of intravenous propofol (Diprivan, AstraZeneca Pharmaceutical, Madrid, Spain), and 0.6 mg/kg of intravenous atracurium (Tracrium, GlaxoSmithKline, Madrid, Spain). Intubation was performed with French tube number 6.5 and a pneumatic tamponade.

Each animal was connected to controlled mechanical ventilation through a ventilator. An inspired fraction of oxygen of 60% and tidal volume of 8-10 ml/kg were administered to maintain normocapnia (end-tidal CO_2_ of 35-40 mmHg). Anesthesia was maintained with fentanyl at a dose of 2.5 *μ*g/kg/h, propofol at 10 mg/kg/h, and atracurium at 0.3 mg/kg/30 min. As a maintenance serum, Ringer's lactate solution was administered at 8 ml/kg/h. The right femoral artery and the right femoral vein were canalized and connected to a PICCO monitor to measure cardiac output.

After these procedures, the animals were placed in right lateral decubitus. A left thoracotomy was performed through the fourth intercostal space with a lower costoctomy, and left pneumonectomy was carried out. When sectioning the left main bronchus, unipulmonary ventilation was initiated with the orotracheal tube progressing to the right main bronchus under control by fiberoptic bronchoscopy. Pulmonary protection strategies were initiated, and the tidal volume was decreased to 6 ml/kg.

Once the left bronchus was sectioned, the pulmonary artery and the caudal pulmonary vein were occluded with forceps, and the cranial pulmonary vein was ligated. The left pulmonary artery was occluded with a protected clamp near the bifurcation of the main pulmonary artery and sectioned distally, leaving a margin of 5 to 10 mm to allow for the arterial anastomosis to be performed in the reimplantation. The cranial pulmonary vein was ligated near the atrium and sectioned. To complete the pneumonectomy, the pulmonary vein of the caudal lobe was clamped near the mouth of the mediastinal lobe vein, sectioned one or two millimeters from the clamp, and sutured with a 6/0 polypropylene continuous stitch. Systemic heparinization with 300 IU/kg (Mayne Pharma Spain) was performed using a bolus prior to the occlusion of the pulmonary artery.

Next, an ex situ cranial lobectomy was performed. The graft was antegradely and retrogradely perfused with a solution from the University of Wisconsin at 10-15°C while ventilating simultaneously with an Ambu bag, at 12 breaths per minute, with ambient air.

Subsequently, the caudal lobe was reimplanted by bronchial-bronchial, arterio-arterial, and veno-auricular anastomoses. The tube was withdrawn into the trachea to allow ventilation of the implant, and reperfusion was started. The reperfusion was maintained for 60 minutes, after which the animal was euthanized by deepening anesthesia and cardioplegia with intravenous potassium chloride.

### 2.2. Study Parameters

Hemodynamic and arterial blood gas studies were carried out at three points: baseline (30 minutes after the anesthetic induction, just before the thoracotomy and with the animal already hemodynamically stabilized and in bipulmonary ventilation), prereperfusion (before reperfusion and ventilation of the left caudal lobe after it has already been reimplanted), and postreperfusion (60 minutes after reperfusion of the left caudal lobe). After 60 minutes of reperfusion, histological samples were collected by lung biopsy of the implanted lobe. Biopsies were preserved in formaldehyde.

For arterial blood gas studies, partial pressure of oxygen (PaO_2_), partial pressure of carbon dioxide (PaCO_2_), and blood pH values were measured from blood samples obtained from the femoral artery. For hemodynamic studies, the mean arterial pressure, heart rate, stroke volume variation, and cardiac output were evaluated using the PICCO system.

To perform histological studies, the samples were fixed in a 10% buffered formaldehyde solution for 24 hours. They were then included in paraffin blocks for subsequent sectioning in 4 *μ*m sections and assembled in histological preparations. The degrees of inflammation, congestion, and edema were studied by hematoxylin-eosin staining.

In samples indicating the presence of inflammatory cells, the presence or absence of cells of the monocyte-macrophage system, neutrophils, or lymphocytes was described. Congestion was defined as the presence of alveolar capillaries or small vessels that were dilated and filled with red blood cells. Edema was defined as thickening of the alveolar or interlobar septa. The three parameters were classified with a quasi-quantitative scale of 0 to 3 (0 absence, 1 scarce presence, 2 moderate presence, and 3 abundant presence) [10].

On the other hand, the expression of markers of inflammation and apoptosis in the histological preparations was determined through immunohistochemical detection. Monoclonal and polyclonal antibodies (Ab) were used against CD68 (1 : 100, MBS370038, MyBioSource), which is an antigen expressed mainly in cells of the monocyte-macrophage system; monocyte chemoattractant protein-1 (MCP-1) (1 : 100, MBS2027425, MyBioSource), which is involved in the inflammatory response; Bcl-2 (1 : 100, 8C8, Novus Biologicals), an antiapoptotic protein; and caspase-9 (1 : 100, orb1677, Biorbyt), a proapoptotic protein. Tissue sections were incubated with hydrogen peroxide after rehydration to water. Then, they were incubated with the serum of the animal in which the secondary antibody was raised in and triton-buffer (Tritón-X100, Sigma-Aldrich). Afterwards, biopsies were incubated with the primary antibody for 24 hours, at 4°C, in the dark. Finally, they were washed in phosphate-buffered saline (0.1 M, pH 7.4) and incubated with biotin conjugated secondary antibody (1 : 200, 1 hour, room temperature, in the dark). All samples were processed, fixed, stained, and imaged in parallel, under the same conditions.

A total of 10 microphotographs were taken at random at 40 magnifications from these immunohistological preparations. All photographs were taken with a Leica DMRB photomicroscope and a Nikon DS-Fi1 camera and cataloged by two observers who were blinded to the treatment group to which the samples belonged.

The 10 images were obtained by starting from the central zone of the histological preparation and moving in a clockwise direction. Each image was used to carry out a descriptive study to detail the type of cell that was positive for any of the antibodies tested in the immunohistochemistry study, the location of the staining (cytoplasmic, nuclear, or mixed), and its intensity in a quasi-quantitative study. In the case of CD68, MCP-1, and Bcl-2 expression studies, two independent observers who were unaware of the origin of the sample counted the numbers of cells marked. If there was a discrepancy, the photo was reviewed, and an agreement was reached. Then, the average of the 10 values was assigned to the sample. Given the broad expression of caspase-9 in some stains, it was measured with a semiquantitative scale (0 absence of staining, 1 > 50% of stained alveolar cells, and 2 < 50% of stained alveolar cells) [11]. The final value was the median of the values obtained.

### 2.3. Statistical Analysis

A database was created using the program IBM SPSS Statistics 24 for Mac. The qualitative and quasi-quantitative variables are expressed as a frequency (percentage). The quantitative ones are presented as mean (typical error) when they follow a normal distribution. The quasi-quantitative variables were treated as qualitative variables and analyzed using a chi-squared test to detect differences between the study groups. The results are expressed in a contingency table. The Kruskal-Wallis test was used to identify significant differences in the quantitative variables between groups. Subsequently, the Mann–Whitney test was used to analyze the pairs of specific samples and to find significant differences. Statistical significance was established with *p* < 0.05.

## 3. Results

### 3.1. Demographic Variables and Surgical Times

In the analysis of the general variables, no statistically significant differences were observed between any of the groups with respect to the weight of the animals (34 (1) CON vs. 39 (2) LIDO vs. 37 (1) kg SHAM, *p* > 0.05) or the duration of the surgical procedure (249 (4) CON vs. 255 (9) LIDO vs. 222 (8) minutes SHAM, *p* > 0.05). No significant differences were found between the CON and LIDO groups in relation to the time of pulmonary ischemia (123 (3) vs. 125 (4) minutes, *p* > 0.05).

### 3.2. Hemodynamic and Arterial Blood Gas Variables (Table 1)

No statistically significant differences were found between the three groups in the comparison of hemodynamic values and the arterial blood gas analysis.

### 3.3. Histopathological Results

#### 3.3.1. Hematoxylin-Eosin Stain

*(1) Tissue Inflammation (Table 2)*. Ischemia-reperfusion induced an increase in inflammatory infiltrate in the lung tissue that was not evident in animals treated with lidocaine (CON vs. SHAM, *p* = 0.026, LIDO vs. CON, *p* = 0.026). The inflammatory analysis by predominant cell type showed that cell infiltration of the monocyte-macrophage strain is more abundant in the group that underwent IRI (CON vs. SHAM, *p* = 0.005). Furthermore, this increase was not evident in the group treated with lidocaine (LIDO vs. CON, *p* = 0.027).

*(2) Interstitial Edema and Capillary Congestion (Table 3)*. No significant differences were found in the degree of pulmonary tissue edema in the different study groups at 60 minutes postreperfusion. IRI induces an increase in congestion in lung tissue, which was higher in the CON group than in the non-ischemia-reperfusion group (CON vs. SHAM, *p* = 0.005). There were no differences in the degree of congestion of the lungs treated with lidocaine and the rest of the groups.

#### 3.3.2. Immunohistochemical Detection (Figure 1)

**Figure 2:**
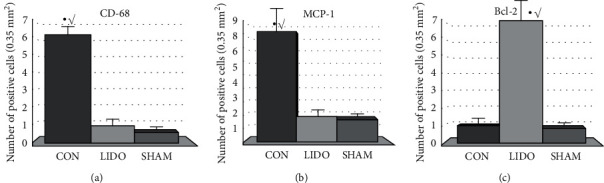
Quantitative analysis of CD68, MCP-1, and Bcl-2 positive cells. Samples were collected 60 minutes after reperfusion. The results are expressed as the average of the number of positive cells found in 10 images of 0.35 mm^2^. CON: control group; LIDO: group treated with lidocaine; SHAM: simulated surgery group. (a) ^∗^*p* = 0.002, CON vs. SHAM. √ *p* = 0.003, CON vs. LIDO. (b) ^∗^*p* = 0.008, CON vs. SHAM. √ *p* = 0.013, CON vs. LIDO. (c) ^∗^*p* = 0.006, LIDO vs. SHAM. √ *p* = 0.008, LIDO vs. CON.

*(1) Expression of CD68 (Figure 2)*. Ischemia-reperfusion induced an increase in the expression of CD68 (6.17 (0.65) vs. 0.16 (0.16), CON vs. SHAM, *p* = 0.002), which was not detected in the lungs that were treated with lidocaine (0.83 (0.31) vs. 6.17 (0.65), LIDO vs. CON, *p* = 0.003). No differences were observed between the LIDO and SHAM groups.

*(2) Expression of MCP-1 (Figure 2)*. Ischemia-reperfusion induced an increase in the expression of MCP-1 (8.25 (2.09) vs. 1.5 (0.58), CON vs. SHAM, *p* = 0.008), which was not noted in the lungs that were treated with lidocaine (2 (0.59) vs. 08.25 (2.09), LIDO vs. CON, *p* = 0.013). No differences were observed between the LIDO and SHAM groups.

*(3) Expression of Bcl-2 (Figure 2)*. In the lungs subjected to ischemia-reperfusion and treated with lidocaine, an increase in the expression of Bcl-2 was observed with respect to the SHAM group (7 (2.24) vs. 1 (0.44), LIDO vs. SHAM, *p* = 0.008) and the CON group (7 (2.24) vs. 1 (0.36), LIDO vs. CON, *p* = 0.008). No differences were observed between the SHAM and CON groups.

**Figure 3:**
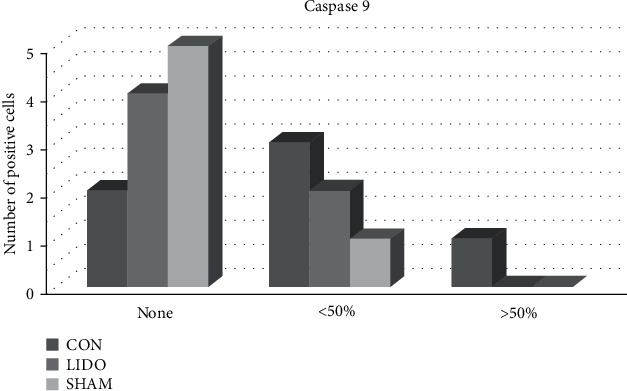
Quasi-quantitative analysis of caspase-9 positive cells. Samples were collected 60 minutes after reperfusion. The results are expressed as the frequency of cases in each group. No significant differences were observed in the caspase-9 expression among groups (*p* > 0.05).

*(4) Expression of Caspase-9 (Figure 3)*. There were no differences in the number of cells that expressed caspase-9 at 60 minutes of reperfusion between the three groups.

## 4. Discussion

In our experimental model of pulmonary autotransplantation, lidocaine attenuated the degree of inflammation of the transplanted lung 60 minutes after reperfusion. Furthermore, it decreased the degree of infiltration by monocyte-macrophage cells, which was observed microscopically in hematoxylin-eosin staining. Although the antiarrhythmic or anesthetic effects of lidocaine are the best known, different experimental and clinical studies have shown its ability to scavenge oxygen radicals and superoxides and inhibit the function of granulocytes [12–16], as well as antiapoptotic effects [17]. Our group has recently demonstrated the anti-inflammatory effects of this drug in an experimental environment that was very similar to the clinical scenario involved in pulmonary lobectomy [8, 9, 18]. However, to our knowledge, no previous investigations have shown what the histological effects of intravenous lidocaine administration are in this experimental model.

Different biomarkers have been used to study induced lung damage inflammation [19–21], including CD68, which is an intracellular glycoprotein that is mainly expressed mainly in tissue macrophages and monocytes. In our study, immunohistochemical staining with anti-CD68 confirmed that ischemia-reperfusion induces an increase in infiltration by cells of the monocyte-macrophage system in lung tissue in the early phase of reperfusion, and the use of intravenous lidocaine inhibited this infiltration. In the animals in which lidocaine was used, we observed a lower infiltration of pneumocytes and monocytic cells expressing MCP-1 in the lungs subjected to IRI. An important relationship has been demonstrated between MCP-1 and primary graft dysfunction in lung transplantation in humans, which proves its prognostic utility [22, 23]. In addition to allowing the transmigration of circulating monocytes to tissues, MCP-1 produces other effects on these cells, such as superoxide anion induction, chemotaxis, and calcium flux [24].

In a model of one-lung IRI in mice, Altemeier et al. [25] used immunohistochemistry analyses and showed an upregulation of MCP-1 and vascular dysfunction in both lungs as a direct result of unilateral IRI. Our group has previously observed that pulmonary IRI presents with an increase in MCP-1, TNF-*α*, and IL-1 and that ischemic preconditioning significantly reduces the expression of all these mediators [26]. Li et al. [27] postulated that lidocaine causes a dose-dependent inhibition of MCP-1 secretion in human monocyte cultures, as well as the suppression of MCP-1 messenger RNA expression induced by lipopolysaccharide. This suggests that MCP-1 inhibition occurs at the transcriptional level. One of the mechanisms proposed to explain the anti-inflammatory effects of LA is the inhibition of the release of chemoattractants by leukocytes, such as MCP-1 [27]. This finding is consistent with those in our experimental study.

Similarly, Sasagawa et al. [28] described the inhibitory effect of lidocaine on the chemotaxis of neutrophils. One of the proposed mechanisms that explain this effect is the inhibition by lidocaine of the accumulation of calcium and a subsequent decrease in the influx of this ion in mast cells [29]. This suggests that it is capable of suppressing the monocytic activation triggered by MCP-1 in the inflammatory process.

The present results complement previously published results, in which intravenous lidocaine in an animal lobectomy model showed a decreased expression of inflammatory and proapoptotic mediators [8]. Subsequent studies have observed a decrease in the production of microRNA related to the pathogenesis of organ rejection in a lung autotransplant model in pigs [9]. This partly explains the anti-inflammatory effects of lidocaine found in this experimental model.

Microvascular dysfunction mediates many of the local and systemic phenomena of IRI damage and especially affects arterioles, capillaries, and venules. In postcapillary venules, the recruitment and transmigration of leukocytes also compromise the integrity of the endothelial barrier and increase oxidative phenomena, thus producing tissue edema [30]. The capacity to reduce edema in the IRI of several organs has been demonstrated by several LAs, including lidocaine [31–34]. Das et al. describe how the intravenous perfusion of lidocaine in isolated lungs subjected to ischemia-reperfusion shows a lower ratio of wet lung/dry lung, lower pulmonary artery pressure, and lower peak pressure [32]. It has also been shown that early postoperative treatment with lidocaine attenuates pulmonary edema [35]. The responsible mechanism seems to be the decrease in the release of leukotrienes by activated human polymorphonuclear cells and monocytes [35–37].

Pulmonary edema is considered the greatest exponent of tissue damage produced by IRI and is not evident in the early phases of ischemia-reperfusion, which were analyzed in the present study. For this reason, we consider this phenomenon to be mainly due to the early time at which the samples were obtained, despite not seeing differences between the degrees of congestion and edema of the lungs treated with lidocaine compared to those that were not treated.

The final result of IRI in the lung is a form of combined cell death, which is characterized by the simultaneous occurrence of necrosis and apoptosis [38]. We observed an increase in the expression of the antiapoptotic protein Bcl-2 in lungs subjected to ischemia-reperfusion, and there were no changes in the expression of the proapoptotic protein caspase-9. Apoptosis can occur through a great variety of different biochemical mechanisms, and the timeline of these biochemical events depends on several factors, such as the cell line, the apoptotic agent, its concentration, the exposure time, and the specific pathway of active apoptosis.

In general, apoptosis induced in cells in culture usually shows signs of apoptosis much earlier (between 5 and 10 hours) than cells in tissues (between 11 and 14 days) [39]. In the field of human lung transplantation, apoptotic phenomena are practically not identified in periods of cold or hot ischemia for up to 5 hours [40]. In an animal lung transplant study, Cooke et al. reported that the overexpression of Bcl-2 attenuates oxidative stress in the transplanted lung, thus decreasing interleukin-1 activity and the activation of the caspase pathway [41].

Lidocaine plays a controversial role in apoptosis. Some suggest that the drug inhibits it, while others report that it induces it. Other studies report lidocaine effectively promotes apoptosis but only in specific cell lines, such as tumor cells rather than healthy tissues [42]. Some literature points out that LAs inhibit proliferation, suppress cell migration, and decrease apoptosis when used in a specific dose range [43].

It can be assumed that the plasma concentrations used in our experiment would be around 5 *μ*g/ml through comparison with other models. The doses used suggest the proapoptotic concentration of lidocaine is one hundred times higher and that the drug exposure times are much longer, which may explain the discrepancy in the results. Studies investigating the antiapoptotic effect use doses within antiarrhythmic lidocaine ranges. In our study, we administered similar doses to those described in similar studies [8]. When studying in vivo models, it is possible that some late apoptotic events are never detected since the apoptotic cells of the tissues are often phagocytosed before such events occur [39]. This could explain why the expression of caspase-9 did not differ between the three study groups, but Bcl-2 did show differences.

In our experimental model of lung transplantation, intravenous lidocaine was associated with an attenuation of the histological markers of lung damage in the early stages of reperfusion. Our current findings suggest that intravenous lidocaine can decrease the inflammatory and apoptotic response in lung IRI, although the biological pathway remains unclear. Further clinical trials are needed in order to acknowledge its effects on ischemia-reperfusion injury in humans. Although challenging, additional studies, with longer reperfusion times and deeper histological assessment, are also warranted.

## Figures and Tables

**Figure 1:**
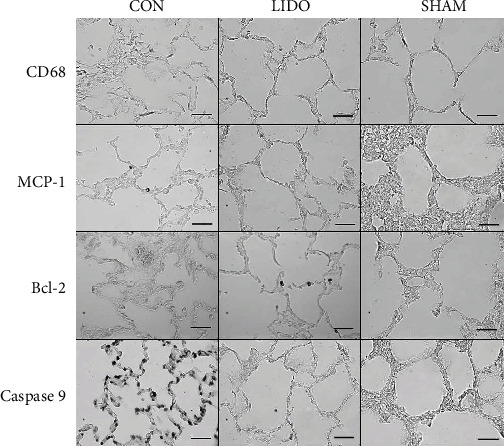
Immunohistochemical detection of CD68, MCP-1, Bcl-2, and caspase-9 in lung biopsies. Samples were collected 60 minutes after reperfusion. Area of peripheral lung parenchyma consisting mostly of well-preserved alveolar wall. Strong nuclear or cytoplasmic staining macrophages or pneumocytes are identified. Images captured with 40x magnification objective (the mark scale corresponds to 30 *μ*m).

**Table 1:** Hemodynamic and blood test results.

	Group	BAS	PRER	POSR60
MAP (mmHg)	CON	91 ± 7	91 ± 4	76 ± 5
	LIDO	92 ± 6	82 ± 8	72 ± 7
	SHAM	93 ± 9	98 ± 6	93 ± 8

HR (bpm)	CON	87 ± 5	94 ± 4	97 ± 7
	LIDO	87 ± 6	88 ± 4	87 ± 5
	SHAM	93 ± 8	90 ± 8	96 ± 4

SVV (%)	CON	12 ± 1	11 ± 1	9 ± 1
	LIDO	17 ± 2	7 ± 1	9 ± 2
	SHAM	15 ± 1	9 ± 1	8 ± 1

PaO_2_ (mmHg)	CON	287 ± 26	205 ± 18	274 ± 65
	LIDO	361 ± 47	222 ± 28	304 ± 22
	SHAM	313 ± 13	264 ± 16	330 ± 10

PaCO_2_ (mmHg)	CON	44 ± 5	37 ± 1	43 ± 3
	LIDO	38 ± 2	39 ± 2	37 ± 2
	SHAM	41 ± 2	44 ± 3	47 ± 4

pH	CON	7.4 ± 0.04	7.5 ± 0.02	7.4 ± 0.03
	LIDO	7.5 ± 0.03	7.5 ± 0.02	7.5 ± 0.03
	SHAM	7.5 ± 0.01	7.4 ± 0.02	7.4 ± 0.02

Results are presented as mean ± typical error. BAS: baseline; PRER: prereperfusion; POSR60: postreperfusion 60 minutes; CON: control group; LIDO: group treated with lidocaine; SHAM: simulated surgery group; MAP: mean arterial pressure; HR: heart rate; SVV: stroke volume variation; PaO_2_: partial pressure of oxygen; PaCO2: partial pressure of carbon dioxide. No significant differences were observed among groups (*p* > 0.05).

**Table 2:** Histopathological assessment of inflammation in ischemia-reperfusion lung injury by hematoxylin-eosin staining. Samples were collected 60 minutes after reperfusion.

Group	Degree	Inflammation (^∗^, #)		Infiltration					
				MM (^∗^, #)		Lymphocytes		Neutrophils	
		*N*	*P*	*N*	*P*	*N*	*P*	*N*	*P*
CON	Absence	1	16.7%	1	16.7%	2	33.3%	6	100%
	Scarce	4	66.7%	5	83.3%	3	50%	0	0%
	Moderate	1	16.7%	0	0%	1	16.7%	0	0%
	Abundant	0	0%	0	0%	0	0%	0	0%

LIDO	Absence	5	83.3%	5	83.3%	5	83.3%	6	100%
	Scarce	1	16.7%	1	16.7%	1	16.7%	0	0%
	Moderate	0	0%	0	0%	0	0%	0	0%
	Abundant	0	0%	0	0%	0	0%	0	0%

SHAM	Absence	5	83.3%	6	100%	5	83.3%	6	100%
	Scarce	1	16.7%	0	0%	1	16.7%	0	0%
	Moderate	0	0%	0	0%	0	0%	0	0%
	Abundant	0	0%	0	0%	0	0%	0	0%

MM: monocyte-macrophage system; *N*: number of cases in the group; *P*: percentage of cases in the group; CON: control group; LIDO: group treated with lidocaine; SHAM: simulated surgery group. ^∗^*p* < 0.05 SHAM vs. CON; #LIDO vs. CON.

**Table 3:** Histopathological assessment of edema and congestion in ischemia-reperfusion lung injury by hematoxylin-eosin staining. Samples were collected 60 minutes after reperfusion.

Group	Degree	Edema		Congestion	
		*N*	*P*	*N*	*P*
CON	Absence	0	0%	1	16.7%
	Scarce	5	83.3%	5	83.3%
	Moderate	1	16.7%	0	0%
	Abundant	0	0%	0	0%

LIDO	Absence	3	50%	4	66.7%
	Scarce	3	50%	1	16.7%
	Moderate	0	0%	1	16.7%
	Abundant	0	0%	0	0%

SHAM	Absence	3	50%	6	100%
	Scarce	3	50%	0	0%
	Moderate	0	0%	0	0%
	Abundant	0	0%	0	0%

*N*: number of cases in the group; *P*: percentage of cases in the group; CON: control group; LIDO: group treated with lidocaine; SHAM: simulated surgery group. The degree of congestion was higher in the CON group than in the non-ischemia-reperfusion group (CON vs. SHAM, *p* = 0.005).

## Data Availability

Data supporting the findings of this study are available from the authors upon request.
